# Genome-wide screens for effective siRNAs through assessing the size of siRNA effects

**DOI:** 10.1186/1756-0500-1-33

**Published:** 2008-06-23

**Authors:** Xiaohua Douglas Zhang

**Affiliations:** 1 Biometrics Research, Merck Research Laboratories, West Point, PA 19486, USA

## Abstract

**Background:**

RNA interference (RNAi) has been seen as a revolution in functional genomics and system biology. Genome-wide RNAi research relies on the development of RNAi high-throughput screening (HTS) assays. One of the most fundamental challenges in RNAi HTS is to glean biological significance from mounds of data, which relies on the development of effective analytic methods for selecting effective small interfering RNAs (siRNAs).

**Findings:**

Based on a recently proposed parameter, strictly standardized mean difference (SSMD), I propose an analytic method for genome-wide screens of effective siRNAs through assessing and testing the size of siRNA effects. Central to this method is the capability of SSMD in quantifying siRNA effects. This method has relied on normal approximation, which works only in the primary screens but not in the confirmatory screens. In this paper, I explore the non-central *t*-distribution property of SSMD estimates and use this property to extend the SSMD-based method so that it works effectively in either primary or confirmatory screens as well as in any HTS screens with or without replicates. The SSMD-based method maintains a balanced control of false positives and false negatives.

**Conclusion:**

The central interest in genome-wide RNAi research is the selection of effective siRNAs which relies on the development of analytic methods to measure the size of siRNA effects. The new analytic method for hit selection provided in this paper offers a good analytic tool for selecting effective siRNAs, better than current analytic methods, and thus may have broad utility in genome-wide RNAi research.

## Findings

### Background

Mean difference, fold change, percent inhibition, percent activity, percent viability, *Z*-score and their robust versions have been used to quantify effect size of an siRNA or a compound in HTS assays [1-7]. However, these metrics have issues in capturing data variability or being affected by sample size and hence cannot effectively assess the size of effect. The *p*-values from the *Z*-score method (or equivalently Mean ± *k *SD and its variant Median ± *k *MAD) and classical *t*-test have widely been used to evaluate the chance of including siRNAs with no specific impact [1,2,5-7]. However, it is mean difference that these methods aim to test, and it is well-known that mean difference cannot effectively measure the magnitude of impact. In addition, the *p*-value from the *Z*-score method or *t*-test is affected by both sample size and the size of siRNA effect.

A recently proposed parameter, strictly standardized mean difference (SSMD) [8], measures the magnitude of impact more effectively than any other currently used metrics. SSMD has been applied for quality control in genome-scale RNAi research [8-10]. Utilizing the fact that SSMD effectively measures the size of effect, Zhang proposes an SSMD-based hit selection method to maintain a balanced control of both FPR and FNR [11]. This method has also been applied to select hits in RNAi HTS primary experiments [12]. However, this method is based on normal approximation, which works only in the primary screens but not in the confirmatory screens. Here I construct a new analytic method for hit selection in HTS assays using non-central *t*-distribution property of SSMD estimates. This method works effectively whether sample size is small or large.

### Issues of hit selection methods in primary screens

**Figure 1:**
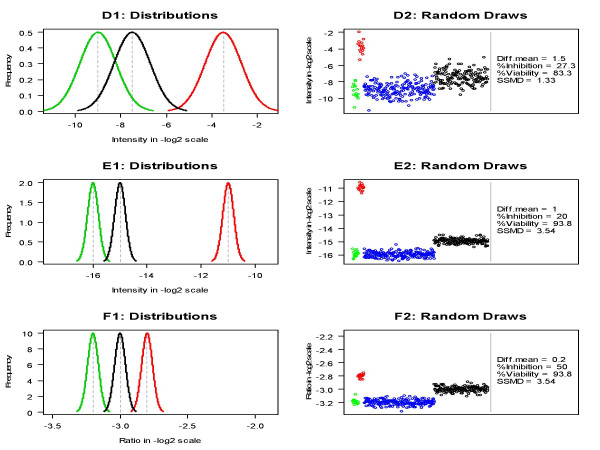
**Comparison of mean difference (Diff.mean), percent inhibition (% Inhibition), percent viability (% Viability) and SSMD in three plates D, E and F**. The population distributions of a positive control (red curve), a sample siRNA (black curve) and a negative control (green curve) in Plates D, E and F are displayed in Panels D1, E1 and F1 respectively. Panels D2, E2 and F2 show 16 random draws from the positive control (red points), 16 draws from the negative control (green points), another 200 draws from the negative control (blue points) and 152 draws from the sample siRNA (black points) in Plates D, E and F respectively.

The size of siRNA effect is commonly assessed using percent inhibition/activation (i.e., $\frac{y-{\bar{X}}_{-}}{{\bar{X}}_{+}-{\bar{X}}_{-}}\times 100$ where y is the measured intensity of an siRNA, ${\bar{X}}_{+}$ is the average intensity of a positive control and ${\bar{X}}_{-}$ the average intensity of a negative reference) and percent viability/activity (i.e., $\frac{y}{{\bar{X}}_{-}}\times 100$) or fold change (i.e., $\frac{y}{{\bar{X}}_{-}}$). Another commonly used method for hit selection in a primary HTS experiment is the *Z*-score method (i.e., $\frac{y-{\bar{X}}_{-}}{s_{-}}$ where *s*_- _is sample standard deviation of a negative reference) along with its variants such as median ± *k *MAD method. The issues of *Z*-score method and its variants have been illustrated in [11,12]. The issues of percent inhibition and percent viability are illustrated in Figure 1. Here we consider the situation where the knockdown of a gene inhibits cell growth. It is clear that the magnitude of difference between the sample siRNA (represented by the black curve) and the negative control (represented by the green curve) is much less in Plate D than in Plates E and F. That is, the siRNA in Plate D has less inhibition effect than in Plates E and F. However, if using percent inhibition, we would conclude that the inhibition effect of the siRNA in Plate D (which has a percent inhibition of 27.3) is larger than the effect of the siRNA in Plate E (which has a percent inhibition of 20). If using percent viability, we would conclude that the inhibition effect of the siRNA in Plate D (which has a percent viability of 83.3) is larger than the effect of the siRNA in Plate E (which has a percent viability of 93.8). Therefore, both percent inhibition and percent viability produce misleading results.

### Issues of hit selection methods in screens with replicates

In all confirmatory HTS screens and some primary screens, there are several sets of source plates. Each set is unique and has replicates (usually triplicates), thus each siRNA has replicates. Because plate-to-plate variability is usually higher than within-plate variability, a paired *t*-test is often used for hit selection in a confirmatory screen. That is, for each siRNA, we calculate the difference between the measured intensity of the siRNA and average intensity of a negative control in a plate, then calculate the corresponding *p*-value of the paired *t*-test in which the null hypothesis of zero mean difference is tested.

**Figure 2:**
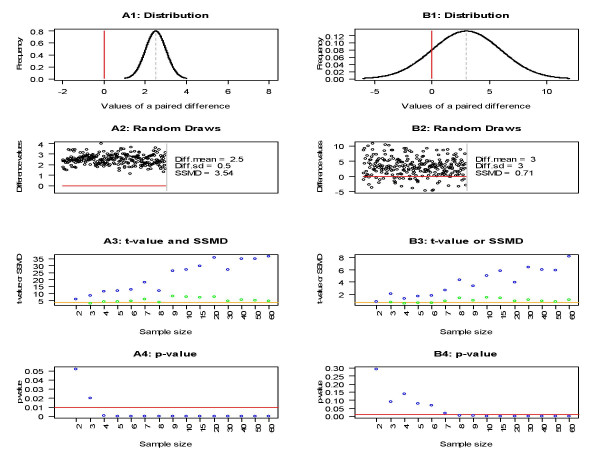
**SSMD, *Z*-score and p-value of random samples of two paired differences corresponding to two siRNAs, siRNA A and siRNA B, respectively**. Panels A1–A4 correspond to siRNA A and Panels B1–B4 correspond to siRNA B. Panels A1 and B1 display the population distributions of paired differences for siRNAs A and B respectively. Panels A2 and B2 show the appearance of random samples of the paired differences for siRNAs A and B respectively. "Diff.mean" and "Diff.sd" denote the mean and standard deviation of a paired difference respectively. In Panels A3 and B3, the blue (or green) points denote the *t*-values (or SSMD estimated values) of samples from siRNA A and siRNA B respectively; the orange lines denote the SSMD population values. In Panels A4 and B4, the blue points denote the *p*-values from t-test of testing paired difference mean and the red lines denote the cutoff of 0.01.

The strength of siRNA impact in a screen with replicates is represented by the magnitude of a paired difference between the measured intensity of an siRNA and average intensity of a negative reference. For one siRNA, a good metric for the assessment of siRNA effect should have one fixed population value and should have estimated values distributed around this population value if it is a statistical parameter. If it is not a statistical parameter, a good metric should have values distributed around a fixed value that can indicate effect size of the siRNA. The *t*-value, *Z*-score and their corresponding *p*-values are not statistical parameters. The *t*-values of the samples from siRNA A are not distributed around a fixed value, and actually go to infinity as sample size increases (blue points in Panel A3 of Figure 2). The corresponding *p*-values go to zero and thus cannot indicate the effect size of the corresponding siRNA (blue points in Panel A4). A similar situation occurs for the *t*-values and *p*-values corresponding to siRNA B (blue points in Panels B3 and B4 of Figure 2).

It is clear that the magnitude of paired difference for siRNA A is much larger than for siRNA B although the mean of the paired difference in Panel A1 (i.e., 2.5) is smaller than that in Panel B1 (i.e., 3). The black points in Panels A2 and B2 (i.e., random draws from the populations in Panels A1 and B1 respectively) also demonstrate that the magnitude of the paired difference in Panel A1 is larger than that in Panel B1. Therefore, a good metric for the assessment of siRNA impact should have a larger (or smaller) value for siRNA A than the value for siRNA B in the case where a large (or small) value of this metric indicates a large effect size. The *t*-value and *p*-value are both affected by sample size; thus we may obtain a larger *p*-value (or smaller *t*-value) corresponding to the samples from siRNA A than from siRNA B. For example, the *p*-values corresponding to the samples with 2 or 3 replicates from siRNA A are larger than the *p*-values corresponding to the samples with at least 10 replicates from siRNA B (Panels A4 and B4 of Figure 2). Therefore, the *p*-value from *t*-test or *Z*-score method cannot effectively measure the strength of siRNA impact.

### Assessment of siRNA effects using SSMD

SSMD is a statistical parameter that measures the magnitude of both paired and unpaired differences and thus can be used to measure the magnitude of impact of siRNAs in both primary and confirmatory screens. For example, the values of *SSMD *between the siRNA and the negative control are 1.33, 3.54 and 3.54 in Plate D, E and F respectively, which appropriately indicates that the effect of the siRNA in Plate D is less than in Plates E and F and that the effect of the siRNA in Plate E is the same as in Plate F (Figure 1). The population values of SSMD for siRNA A and siRNA B are 3.54 and 0.71 respectively (Figure 2). The estimated SSMD values (denoted by the green points) all fall around the population values of SSMD (denoted by the orange lines) and do not have an increasing trend as sample size increases (Panels A3 and B3 of Figure 2). All these results indicate that SSMD appropriately indicates the effect size of an siRNA, better than percent inhibition/viability and *p*-value from *t*-test of testing no mean difference.

Based on both original and probability meanings of SSMD, an SSMD-based 1-2-3 rule [11], along with its extended version, has been proposed for classifying siRNA impact. The SSMD-based 1-2-3 rules provide a guideline for classifying the strength of siRNA impact. For example, in Figure 1, the siRNA in Plate D is classified as "moderate inhibition effect" and the siRNAs in Plates E and F are both classified as "strong inhibition effect". In Figure 2, siRNAs A and B are classified as "strong inhibition effect" and "weak inhibition effect" respectively. The 1-2-3 rule and extended 1-2-3 rule work in the situation where the population value of SSMD is known; they also work reasonably when sample size is large. In practice, the population value of SSMD is unknown and sample size is small especially in confirmatory RNAi HTS experiments. In such a case, we can provide a point estimate and a confidence interval of SSMD for each siRNA based on its estimated SSMD value [see additional file 1].

### A balanced control of false positives and false negatives

**Figure 3:**
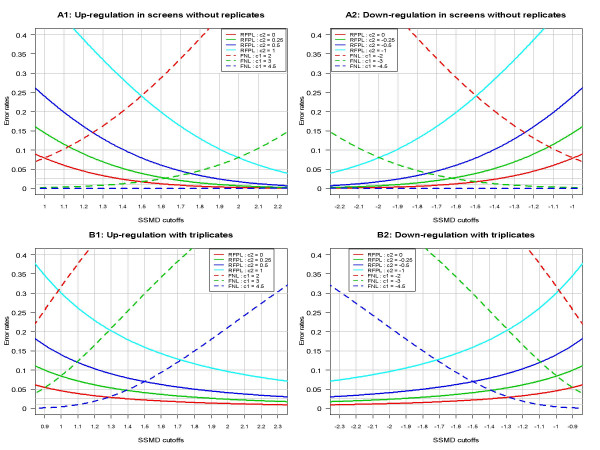
**Error-cutoff plots for controlling both restricted false positive level (RFPL) and false negative level (FNL) in RNAi HTS assays**. Panels A1 and A2 are for primary screens without replicates and Panels B1 and B2 are for confirmatory screens with triplicates. The unlabeled grey horizontal lines in the bottom of each panel indicate error rates of 0.025 and 0.01, respectively.

Based on SSMD, we may maintain a flexible and balanced control of both the false negative rate (FNR), in which the siRNAs with strong effects are not selected as hits, and the restricted false positive rate (RFPR), in which the siRNAs with weak or no effects are selected as hits. The maximum RFPR and FNR in a decision rule are called restricted false positive level (RFPL) and false negative level (FNL), respectively. To use the SSMD-based method for selecting hits in the direction of positive values in HTS assays, we need to search for a cutoff β* for the estimated SSMD so that we can maintain a balanced control of both RFPR and FNR when we use the decision rule of declaring an siRNA as a hit if it has $\hat{\beta }$ ≥ β* and as a non-hit otherwise. The search of a cutoff can be achieved through error-cutoff plots in which we plot RFPLs and FNLs versus cutoffs of estimated SSDM values, as shown in Figure 3.

Based on Table 1, we calculate RFPL and FNL corresponding to each set of values for β*, *c*_1 _and *c*_2_. In a primary screen, the majority of sample wells may be used as the negative reference in a plate. In such a case, *n*_2 _is about 300. To select hits in the direction of positive values, we calculate RFPLs with respect to (w.r.t.) *c*_2 _= 0, 0.25, 0.5, 1 and FNLs w.r.t. *c*_1 _= 2, 3, 4.5 (Panel A1 of Figure 3). The commonly used error rates are 0.05, 0.025 and 0.01 in one direction. From Panel A1 of Figure 3, a cutoff between 1.4 and 1.8 can control RFPL w.r.t. *c*_2 _= 0 to be less than 0.025, RFPL w.r.t. *c*_2 _= 0.25 to be less than 0.051 and FNL w.r.t. *c*_1 _= 3 to be less than 0.05. A cutoff between 1.9 and 2.1 can control RFPL w.r.t *c*_2 _= 0 to be less than 0.005, RFPL w.r.t *c*_2 _= 0.25 to be less than 0.01, and FNL w.r.t. *c*_1 _= 3 to be less than 0.10. Therefore, any cutoff between 1.4 and 2.1 for SSMD is theoretically reasonable and maintains a balanced control of RFPR and FNR for selecting hits in the direction of positive values. Similarly, to select hits in the direction of negative values, a reasonable cutoff is between -2.1 and -1.4 when we use the decision rule of declaring an siRNA as a hit if it has $\hat{\beta }$ ≤ β* and as a non-hit otherwise (Panel A2 of Figure 3). Similarly from Panels B1 and B2 of Figure 3, in confirmatory screens with triplicates, a reasonable cutoff is between 1 and 1.4.

**Table 1:** SSMD-based decision rules and their false negative levels (FNLs) and restricted false positive levels (RFPLs) for hit selection in RNAi HTS experiments

**I: Select up-regulated siRNAs (***c*_1 _≥ *c*_2 _≥ 0**)**		
Selection Criterion	FNL	RFPL

Ia: $\hat{\beta }$ ≥ β*	${\text{F}}_{t(\nu ,bc_{1})}\left(\frac{\beta *}{k}\right)$	$\text{1}-{\text{F}}_{t(\nu ,bc_{2})}\left(\frac{\beta *}{k}\right)$
Ib: $\hat{\beta }\geq kQ_{t(\nu ,bc_{1})}(\alpha_{1})$	*α*_1_	$\text{1}-{\text{F}}_{t(\nu ,bc_{2})}\left(Q_{t(\nu ,bc_{1})}(\alpha_{1})\right)$
Ic: $\hat{\beta }\geq kQ_{t(\nu ,bc_{2})}(1-\alpha_{2})$	${\text{F}}_{t(\nu ,bc_{1})}\left(Q_{t(\nu ,bc_{2})}(1-\alpha_{2})\right)$	*α*_2_

**II: Select down-regulated siRNAs (***c*_1 _≤ *c*_2 _≤ 0**)**		

Selection Criterion	FNL	RFPL

IIa: $\hat{\beta }$ ≤ β*	$\text{1}-{\text{F}}_{t(\nu ,bc_{1})}\left(\frac{\beta *}{k}\right)$	${\text{F}}_{t(\nu ,bc_{2})}\left(\frac{\beta *}{k}\right)$
IIb: $\hat{\beta }\leq kQ_{t(\nu ,bc_{1})}(1-\alpha_{1})$	*α*_1_	${\text{F}}_{t(\nu ,bc_{2})}\left(Q_{t(\nu ,bc_{1})}(1-\alpha_{1})\right)$
IIc: $\hat{\beta }\leq kQ_{t(\nu ,bc_{2})}(\alpha_{2})$	$\text{1}-{\text{F}}_{t(\nu ,bc_{1})}\left(Q_{t(\nu ,bc_{2})}(\alpha_{2})\right)$	*α*_2_

Notes:

(i) $\hat{\beta }$ is the estimate of SSMD and β* is a cutoff of SSMD; $\hat{\beta }$ = *kT *where *T *has a noncentral *t*-distribution with degree of freedom *ν *and non-central parameter *bβ*, namely *T *~*t*(*ν, bβ*); F_*t*(*ν, bβ*) _(·) and *Q*_*t*(*ν, bβ*) _(*α*) are the cumulative distribution function and the *α *quantile of *t*(*ν, bβ*) respectively.

(ii) For an unpaired difference, $\hat{\beta }=\frac{{\bar{X}}_{1}-{\bar{X}}_{2}}{\sqrt{\frac{2}{K}\left((n_{1}-1)s_{1}^{2}+(n_{2}-1)s_{2}^{2}\right)}}$ and $T=\frac{\left({\bar{X}}_{1}-{\bar{X}}_{2}\right)/\sqrt{\frac{1}{n_{1}}+\frac{1}{n_{2}}}}{\sqrt{\left((n_{1}-1)s_{1}^{2}+(n_{2}-1)s_{2}^{2}\right)/\left(N-2\right)}}$ where $K=2\cdot {\left(\frac{\Gamma (\frac{N-2}{2})}{\Gamma (\frac{N-3}{2})}\right)}^{2}\approx N-3.5$, *N *= *n*_1 _+ *n*_2_, and *n*_1_, ${\bar{X}}_{1}$, *s*_1_, *n*_2_, ${\bar{X}}_{2}$, *s*_2 _are sample size, mean and standard deviation in two groups respectively; $k=\sqrt{\frac{K}{2(N-2)}(\frac{1}{n_{1}}+\frac{1}{n_{2}})}$, *ν *= *N *- 2, $b=\frac{\sqrt{2}}{\sqrt{\frac{1}{n_{1}}+\frac{1}{n_{2}}}}$.

(iii) For a paired difference, $\hat{\beta }=\frac{\Gamma (\frac{n-1}{2})}{\Gamma (\frac{n-2}{2})}\sqrt{\frac{2}{n-1}}\frac{\bar{D}}{s_{D}}$ and $T=\frac{\sqrt{n}\bar{D}}{s_{D}}$ where *n*, $\bar{D}$ and *s*_*D *_are sample size, sample mean and standard deviation of a paired difference respectively; $k=\frac{\Gamma (\frac{n-1}{2})}{\Gamma (\frac{n-2}{2})}\sqrt{\frac{2}{n(n-1)}}$, *ν *= *n *- 1, $b=\sqrt{n}$.

The choice of an exact cutoff between 1.4 and 2.1 (or between -2.1 and -1.4) in a real primary experiment relies on the refined tolerance of false positives and false negatives and the capacity of follow-up studies after that experiment. For example, if one has a low tolerance in missing hits with SSMD greater than 2 or 3 (or less than -2 or -3), one may choose a cutoff between 1.4 and 1.6 (or between -1.6 and -1.4). On the other hand, if follow-up studies have a low capacity of including selected hits, one may choose a cutoff between 1.8 and 2.1 (or between -2.1 and -1.8). These cutoffs may maintain a balanced control of both RFPR for including siRNAs with weak or no effects and FNR for excluding siRNAs with strong effects.

## Discussion

SSMD is usually applied to the measured intensity of each siRNA individually. In some screens, there may be a need to pool multiple measured values to a single value. For example, in the situations where there are two or more wells for each siRNA in a plate, we may use the mean or median of these replicates to represent the measured intensity of this siRNA. In screens where multiple siRNAs are designed to target the same gene to account for off-target effects, there may be a need to pool information across these siRNAs to form a single value for a gene. In those situations, SSMD can be applied to the pooled value for either an siRNA or a gene especially when the pooled value has a symmetric or nearly normal distribution.

## Competing interests

The authors declare that they have no competing interests.

## Authors' contributions

XDZ proposed all methods, derived all mathematical formulas, conducted all simulations and drafted the final manuscript.

## Supplementary Material

Additional file 1 SSMD estimate and its distribution. In this file, I provide statistical estimation and confidence interval for both unpaired and paired SSMD, derive non-central t-distribution property of SSMD estimates, and explore false positive and false negative rates when SSMD is used for hit selection in RNAi high-throughput screening experiments. Click here for file
