# Supplementary material for: Genome-wide screens for effective siRNAs through assessing the size of siRNA effects
Source: BMC Res Notes. 2008 Jun 23;1:33. doi: 10.1186/1756-0500-1-33 (PMC2526086; doi:10.1186/1756-0500-1-33)
Supplement: Additional file 1 — SSMD estimate and its distribution. In this file, I provide statistical estimation and confidence interval for both unpaired and paired SSMD, derive non-central t-distribution property of SSMD estimates, and explore false positive and false negative rates when SSMD is used for hit selection in RNAi high-throughput screening experiments. [file 1756-0500-1-33-S1.doc]

**Methods**

**Estimation of unpaired SSMD**

SSMD has recently been proposed for measuring the magnitude of difference between two populations [1]. Let random variables *P_1_* and *P_2_* denote two populations of interest and *D* denote the difference between *P_1_* and *P_2_*. Suppose *P_1_* has mean $\mu_{1}$ and variance $\sigma_{1}^{2}$, and *P_2_* has mean $\mu_{2}$ and variance $\sigma_{2}^{2}$. The covariance between these two populations is $\sigma_{12}$. Then SSMD (denoted as$\beta$) is defined as the ratio of mean $\mu_{D}$ to standard deviation $\sigma_{D}$ of the difference *D*, namely . When two populations are independent, we are interested in unpaired difference between the two populations. The SSMD corresponding to unpaired difference is called “unpaired SSMD”, which is . If the two independent populations have equal variances (namely $\sigma_{1}^{2}=\sigma_{2}^{2}=\sigma^{2}$), then .

SSMD defined above is a population parameter which needs to be estimated from observed samples. Suppose we have one sample (with sample size $n_{1}$, sample mean $\bar{X}_{1}$ and sample standard deviation $s_{1}$) from Population *P_1_* and another independent sample (with $n_{2}$, $\bar{X}_{2}$ and $s_{2}$) from Population *P_2_*. Let N = *n_1_* + *n_2_*. Zhang [1] derived maximum-likelihood estimate (MLE) and method-of-moment (MM) estimate of unpaired SSMD when the two compared groups have normal distributions with unequal variances. When the two compared groups have normal distributions with equal variance, the uniformly minimal variance unbiased estimate (UMVUE) of unpaired SSMD [2] is,

, when $n_{1}\geq2,n_{2}\geq2$.

It is well-known that if two random variables *X* and *U* are independently distributed with $X\sim N(\mu,\text{ }1)$ and $V\sim\chi^{2}(p)$, then the ratio has a noncentral t-distribution with *p* degrees of freedom and noncentrality parameter $\mu$. We know that and , namely .

Therefore, ,

namely .

In primary HTS experiments, $n_{1}=1$ for most investigated siRNAs. $s_{1}^{2}$ does not exist when $n_{1}=1$. In this case, the UMVUE of unpaired SSMD is then where . We know that and , namely .

Therefore, .

If set $(n_{1}-1)s_{1}^{2}=0$ when $n_{1}=1$, then for both $n_{1}=1$ and $n_{1}\geq2$ (i.e., $n_{1}\geq1$),

and

where , $\nu=N-2$, and .

**Estimation of paired SSMD**

When two populations are correlated, we are usually interested in paired difference. The SSMD corresponding to paired difference is called “paired SSMD”. Suppose we observe *n* pairs of samples, $(X_{11},X_{21}),$ $(X_{12},X_{22}),$ $(X_{1n},X_{2n})$ from populations *P_1_* and *P_2_* respectively. Let *D_j_* be the difference between the *j*^th^ pair of samples, namely $D_{j}=X_{1j}-X_{2j}$. Let $\bar{D}$and $s_{D}$ be the sample mean and sample standard deviation of *D* respectively, namely and . Assume that *D* is normally distributed, namely $D\sim N(\mu_{D},\sigma_{D}^{2})$. Then the MM, MLE and UMVUE of the paired SSMD , and respectively. The proof of ML and MLE is trivial. The proof of UMVUE is as follows.

When $D\sim N(\mu_{D},\sigma_{D}^{2})$, there are the following properties: $(\bar{D},s_{D}^{2})$ is a complete sufficient statistic of $(\mu_{D},\sigma_{D}^{2})$; $\bar{D}$ and $s_{D}^{2}$ are independent with each other; and $\frac{(n-1)s_{D}^{2}}{\sigma_{D}^{2}}$ is distributed to $\chi^{2}(n-1)$. Based on these properties, we have

and

.

Set . Then $\hat{\beta}$ is a function of the complete sufficient statistic $(\bar{D},s_{D}^{2})$ and $\hat{\beta}$ is an unbiased estimate of $\beta$. Thus, $\hat{\beta}$ is a UMVUE of $\beta$.

$D_{j}$’s are independently distributed with $N(\mu_{D},\sigma_{D}^{2})$, so and , namely . Therefore,

, namely .

Let . Then $T\text{\textasciitilde noncentral }t(\nu,b\beta)$ and $\hat{\beta}_{UMVUE}=kT$ where $\nu=n-1$, $b=\sqrt{n}$ and .

**Confidence interval of SSMD estimates**

Based on the estimates of SSMD and their distributions derived above, we have $T\text{\textasciitilde noncentral }t(\nu,b\beta)$ and $\hat{\beta}_{UMVUE}=kT$ for both unpaired and paired SSMDs although $\nu=n_{1}+n_{2}-2$, and in unpaired SSMD and $\nu=n-1$, $b=\sqrt{n}$ and in paired SSMD. Let $F_{t(\nu,b\beta)}(\cdot)$ be the cumulative distribution function of $\text{noncentral }t(\nu,b\beta)$ and $T_{\text{obs}}$ be the observed value of $T$. Because $T\sim\text{noncentral }t(\nu,b\beta)$, we can find $\beta_{L}$ and $\beta_{U}$ such that and . Then ($\beta_{L}$, $\beta_{U}$) is a $1-\alpha$ confidence interval of SSMD. The variance of a noncentral $t(\nu,\mu)$ is . Thus, the variance of $T$ is . Using $\hat{\beta}_{UMVUE}=\text{ }kT$, the variance of $\hat{\beta}_{UMVUE}$ is .

**False negative rate and restricted false positive rate**

Let us focus on the situation where we want to select siRNAs with large positive effects, namely the siRNAs with $\beta\geq c_{1}$ where $\beta$ denotes SSMD and $c_{1}$ is the preset lowest value for large effects. In this situation, the FNR is the probability that we conclude $\beta<c_{1}$ whereas actually $\beta\geq c_{1}$. The maximum FNR in a decision rule is called false negative level (FNL). Traditionally, the false positive rate is the probability that we conclude $\beta\geq c_{1}$ whereas actually $\beta<c_{1}$. However, in RNAi HTS experiments, scientists are usually interested in controlling the probability of concluding $\beta\geq c_{1}$ given $\beta\leq c_{2}$ where $0<c_{2}\leq c_{1}$. This probability is called restricted false positive rate (RFPR) [2, 3]. The maximum RFPR in a decision rule is called restricted false positive level (RFPL).

For example, for an observed SSMD value $\beta_{\text{obs}}$ ($\beta_{\text{obs}}>0$), if we select all the siRNAs with $\hat{\beta}\geq\beta_{\text{obs}}$ as hits, the FNR in this process (for $c_{1}\geq c_{2}>0$) is

and the RFPR in this process is

; thus, FNL and RFPL in this process are and respectively. Similarly, for an observed SSMD value $\beta_{\text{obs}}$ ($\beta_{\text{obs}}<0$), if we select all the siRNAs with $\hat{\beta}<\beta_{\text{obs}}$ as hits, FNL and RFPL in this process (for $c_{1}\leq c_{2}<0$) are and respectively. Consequently, when we use SSMD-based ranking method for selecting siRNAs with a large positive value, in the process that we select all the *m* siRNAs with $\hat{\beta}\geq\beta*$ ($\beta*>0$) as hits, the FNL and RFPL are and respectively; when we use SSMD-based ranking method for selecting siRNAs with a large negative value, in the process that we select all the *m* siRNAs with $\hat{\beta}\leq\beta*$ ($\beta*<0$) as hits, FNL and RFPL are and respectively (Selection Criteria Ia and IIa in Table 1).

**Hit selection using SSMD-based testing methods**

Based on $T\text{\textasciitilde noncentral }t(\nu,b\beta)$ and $\hat{\beta}=kT$, we can determine a selection criterion so that a specific FNL or RFPL can be achieved. To select siRNAs with large positive effects, namely the siRNAs with $\beta\geq c_{1}$ ($c_{1}>0$), the following decision rule (namely Selection Criterion Ib in Table 1) achieves FNL to be $\alpha$.

, where $Q_{t(\nu,bc_{1})}(\alpha_{1})$ is the $\alpha_{1}$ quantile of $t(\nu,bc_{1})$. The reason is as follows. The FNR for Decision Rule Ib is the probability that $\hat{\beta}<kQ_{t(\nu,bc_{1})}(\alpha_{1})$ (i.e., not declaring a hit) given $\beta\geq c_{1}$. Hence,

Therefore, $FNL=\alpha$ when using Selection Criterion Ib.

Using Decision Rule Ib, the RFPR with respect to (w.r.t.) $c_{2}$ and $c_{1}$ $(0\leq c_{2}\leq c_{1})$ is

, where $\text{F}_{t(\nu,bc_{2})}\left(\cdot\right)$ is the cumulative distribution function of $t(\nu,bc_{2})$. Therefore, $RFPL=1-F_{t(\nu,bc_{2})}\left(Q_{t(\nu,bc_{1})}(\alpha_{1})\right)$ when using Selection Criterion Ib. Similarly, we obtain Selection Criteria Ic, IIb and IIc and their FNLs and RFPLs listed in Table 1.

References

1. Zhang XD: **A pair of new statistical parameters for quality control in RNA interference high-throughput screening assays**. *Genomics* 2007, **89:**552-561.

2. Zhang XD: **A new method with flexible and balanced control of false negatives and false positives for hit selection in RNA interference high-throughput screening assays**. *Journal of Biomolecular Screening* 2007, **12:**645-655.

3. Zhang XD, Ferrer M, Espeseth AS, Marine SD, Stec EM, Crackower MA, Holder DJ, Heyse JF, Strulovici B: **The use of strictly standardized mean difference for hit selection in primary RNA interference high-throughput screening experiments**. *Journal of Biomolecular Screening* 2007, **12:**497-509.
